# Psychometric properties of the psychosocial screening instrument for physical trauma patients (PSIT)

**DOI:** 10.1186/s12955-019-1234-6

**Published:** 2019-11-12

**Authors:** Maria Karabatzakis, Brenda Leontine Den Oudsten, Taco Gosens, Jolanda De Vries

**Affiliations:** 1 Trauma TopCare, ETZ Hospital (Elisabeth-TweeSteden Ziekenhuis), Tilburg, The Netherlands; 2 Center of Research on Psychological and Somatic Disorders (CoRPS), Department of Medical and Clinical Psychology, Tilburg University, Tilburg, The Netherlands; 3 Department of Orthopaedics and Traumatology, ETZ Hospital (Elisabeth-TweeSteden Ziekenhuis), Tilburg, The Netherlands; 4 Department of Medical Psychology, ETZ Hospital (Elisabeth-TweeSteden Ziekenhuis), P.O. Box 90151, 5000 LC Tilburg, The Netherlands

**Keywords:** Physical trauma, Injury, Psychosocial problems, Screening instrument, Reliability, Validity

## Abstract

**Background:**

Early detection of psychosocial problems post-injury may prevent them from becoming chronic. Currently, there is no psychosocial screening instrument that can be used in patients surviving a physical trauma or injury. Therefore, we recently developed a psychosocial screening instrument for adult physical trauma patients, the PSIT. The aim of this study was to finalize and psychometrically examine the PSIT.

**Methods:**

All adult (≥ 18 years) trauma patients admitted to a Dutch level I trauma center from October 2016 through September 2017 without severe cognitive disorders (*n* = 1448) received the PSIT, Impact of Events Scale-Revised (IES-R), Patient Health Questionnaire-9 (PHQ-9), Rosenberg Self-Esteem Scale (RSES), State-Trait Anxiety Inventory-State (STAI-S), and the World Health Organization Quality of Life-Abbreviated version (WHOQOL-Bref). After 2 weeks, a subgroup of responding participants received the PSIT a second time. The internal structure (principal components analysis, PCA; and confirmatory factor analysis, CFA), internal consistency (Cronbach’s alpha, α), test-retest reliability (Intraclass Correlation Coefficient, ICC), construct validity (Spearman’s rho correlations), diagnostic accuracy (Area Under the Curve, AUC), and potential cut-off values (sensitivity and specificity) were examined.

**Results:**

A total of 364 (25.1%) patients participated, of whom 128 completed the PSIT again after 19.5 ± 6.8 days. Test-retest reliability was good (ICC = 0.86). Based on PCA, five items were removed because of cross-loadings ≥ 0.3. Three subscales were identified: (1) Negative affect (7 items; α = 0.91; AUC = 0.92); (2) Anxiety and Post-Traumatic Stress Symptoms (4 items; α = 0.77; AUC = 0.88); and (3) Social and self-image (4 items; α = 0.79; AUC = 0.92). CFA supported this structure (comparative fit index = 0.96; root mean square error of approximation = 0.06; standardized rood mean square residual = 0.04). Four of the five a priori formulated hypotheses regarding construct validity were confirmed. The following cut-off values represent maximum sensitivity and specificity: 7 on subscale 1 (89.6% and 83.4%), 3 on subscale 2 (94.4% and 90.3%), and 4 on subscale 3 (85.7% and 90.7%).

**Conclusion:**

The final PSIT has good psychometric properties in adult trauma patients.

## Background

Each year, injuries resulting from physical trauma cause worldwide over five million deaths [1]. Tens of millions of people survive an injury and may be confronted with physical or psychosocial problems due to trauma [1]. Between 25% [2]–76% [3] of patients report psychosocial problems as early as 2 weeks after injury. In addition, 7% [4]–25% [5] has psychiatric comorbidity between 3 and 12 months following injury. It is important to recognize psychosocial problems post-injury, since such problems may negatively impact physical recovery [6, 7] and patients’ quality of life (QoL) [8–13]. Psychosocial screening not only prevents problems from escalating, but may also improve communication between patients and health care providers (HCPs) and is time saving because the information provided by screening creates the opportunity to focus on issues that are important for patients [14]. Systematic screening may assist in early detection of psychosocial problems and has received much attention in oncological care [15–17], but not yet in trauma care. Furthermore, there is no psychosocial screening instrument currently available for an adult trauma population. Existing screening instruments are specifically developed for and validated among cancer patients [15–17]. Some of those questionnaires also measure physical problems [17], which may interfere with the detection of psychosocial problems [18]. Therefore, a psychosocial screening instrument should preferably only contain psychosocial problems. Existing questionnaires that are sometimes used in clinical practice mainly focus on psychological problems such as depressive and anxiety symptoms (e.g., the Hospital Anxiety and Depression Scale [19]) or post-traumatic stress symptoms (PTSS) (e.g., the Impact of Events Scale [20]). Yet, injured patients may also experience other psychosocial problems, such as impaired social life [21].

Recently, the Psychosocial Screening Instrument for physical Trauma patients (PSIT) was developed, a self-report instrument which screens for several psychosocial problems after injury. To develop the PSIT, first a systematic review was conducted to generate a comprehensive list of psychosocial problems following physical trauma (submitted). Second, focus groups with trauma patients and HCPs were organized to ask patients which psychosocial problems they experienced and to ask patients and HCPs feedback on the problems list resulting from the review and which problems they perceived as most important (submitted). Whereas studies most frequently have assessed symptoms of depression, post-traumatic stress, and anxiety [22–24], our systematic review and focus groups revealed that trauma patients can experience these but also other psychosocial problems following their trauma, such as a decreased self-esteem [25] and sexual problems [26]. Therefore, these problems were also included in the preliminary version of the PSIT. The aim of this study was to finalize the PSIT and examine its psychometric properties.

## Method

### Participants

Patients were eligible if they were 18 years or older and admitted to a ward or the Intensive Care Unit (ICU) of the ETZ Hospital, a level I trauma center in the Netherlands, from October 2016 to September 2017. Patients were invited using the Brabant Trauma Registry (BTR) database. Exclusion criteria were (i) severe cognitive impairment (e.g., dementia) and (ii) insufficient knowledge of the Dutch language. The Medical Ethical Committee Brabant approved the study. The data were collected between October 2017 and March 2018.

### Procedure

Eligible participants received written explanation about the study and contact details of one of the researchers. When a patient was willing to participate, he/she was asked to sign an informed consent form, complete the questionnaires, and return all documents together in a return envelope. Patients who did not return the questionnaires were called to remind them of the study and, if they were unreachable, they received a reminder by post. After approximately 2 weeks, patients who completed the first set of questionnaires were sent the PSIT again, with a request to complete this instrument a second time to establish test-retest reliability. This approach was chosen because a smaller sample size is needed to examine test-retest reliability compared with other psychometric properties [27]. Participation was voluntarily.

### Measures

#### Demographic and clinical information

The following variables were derived from the patient database: sex, date of birth, date of hospital admission, injury cause, injury mechanism, injury severity score (ISS), and whether they were admitted to the ICU. Patients were asked to provide the following demographic information: level of education, living situation (e.g., alone or with a partner), and whether they currently had a paid job (yes/no). Furthermore, to gain insight in pre-existing psychosocial problems, patients were asked whether they experienced psychological problems before the trauma (yes/no) and if they could briefly describe those problems (if applicable), and if they ever received counseling for psychological problems (yes/no). In addition, patients were asked if they currently received counseling for psychological problems (yes/no).

#### Psychosocial screening instrument for physical trauma patients (PSIT)

The PSIT is a recently developed Dutch psychosocial screening instrument for adult trauma patients. The preliminary PSIT consists of 20 items and covers the following topics: anxiety symptoms (2 items), mood disturbances (2 items), sexual problems (1 item), impaired body image (1 item), loneliness (1 item), feeling burdensome to others (1 item), inadequate social support (1 item), decreased self-confidence (1 item), employment-related problems (1 item), post-traumatic stress symptoms (3 items), impairments in social activities/leisure time (1 item), frustration (1 item), disappointment (1 item), powerlessness (1 item), anger (1 item), and relationship issues (1 item). This preliminary version of the PSIT ended with an open-ended question to provide patients the opportunity to indicate any other psychosocial problem or problems that they experienced. Each item can be answered on a 4-point Likert scale from 0 (*not at all*) to 3 (*very much*). After completion of the PSIT, patients were asked whether they found one or more items confusing or difficult (if yes, which and why), whether they missed a topic (if yes, which topic), and whether they had any remarks about the PSIT.

#### Patient health questionnaire-9 (PHQ-9)

The PHQ-9 is a 9-item measure to assess depressive symptoms. It is considered a suitable questionnaire to screen for depressive symptoms following injury [28]. Each symptom can be rated from 0 (*not at all*) to 3 (*nearly every day*) [29]. The total score ranges from 0 to 18. A score of at least 10 is indicative of depressive symptoms [30–33]. The PHQ-9 has shown good psychometric properties in several trauma populations [30, 31, 34, 35].

#### Impact of events scale-revised (IES-R)

The IES-R consists of 22 items and measures three symptom clusters of PTSS, namely intrusive, avoidance and hyperarousal symptoms [36]. Each symptom can be rated from 0 (*not at all*) to 4 (*extremely*). Scores can range from 0 to 88 and a score of 33 or higher represents the most appropriate cut-off value of PTSS [37]. Studies in several trauma populations have shown good psychometric properties [20, 37, 38].

#### State-trait anxiety inventory - state anxiety subscale (STAI-S)

The STAI-S is a 20-item questionnaire which measures state anxiety [39]. Each item ranges from 1 (*almost never*) to 4 (*almost always*). Despite limited research on useful cut-off values, a score of 40 or higher has been reported to reflect anxiety symptoms [40, 41]. Studies have shown that the STAI is a reliable instrument in several populations [39, 41]. 

#### Rosenberg self-esteem scale (RSES)

The RSES has 10 items and is a self-report instrument to assess global self-esteem [42]. Responses range from 1 (*strongly disagree*) to 4 (*strongly agree*). Although it has been stated that scores should preferably be analyzed in a continuous manner, scores below 15 reflect low self-esteem [43]. The RSES has good psychometric properties [42].

#### World Health Organization quality of life assessment instrument - Bref (WHOQOL-Bref)

The WHOQOL-Bref consists of 26 items and is the short form of the WHOQOL-100 which is developed to assess QoL [44]. Scores are calculated for one facet (Overall QoL and general health) and four domains (Physical Health, Psychological Health, Social Relationships, and Environment) [45]. Higher scores indicate good QoL [46]. The WHOQOL-Bref is a valid and reliable measure to assess QoL in patients with TBI [47] and SCI [48].

### Sample size

Several recommendations exist regarding the minimum sample size needed to assess psychometric properties of an instrument [27, 49, 50]. Studies using Monte Carlo simulations revealed that a minimum of 300 participants is required for exploratory studies [50]. Specifically, to reach good test-retest reliability (i.e., intraclass correlation coefficient or ICC ≥ 0.80), a minimum sample size of 50 is advised [27]. To obtain a representative sample and to account for drop-out, we aimed to include at least 80 patients for the test-retest analysis.

### Statistical analyses

To compare responders and non-responders on demographic and clinical characteristics, chi-squared and Mann-Whitney U tests were calculated. Descriptive statistics were used to create an overview of the sample characteristics. The distribution of item scores on the PSIT was explored with regard to kurtosis and skewness and by performing frequency analyses. Moreover, the presence of floor and ceiling effects was assessed using frequency analyses. Next, principal components analysis (PCA) was used to examine the internal structure of the PSIT. Appropriateness of PCA was checked using the Kaiser-Meyer-Olkin measure (KMO), which should be at least 0.06, and by Bartlett’s test of sphericity, which should be statistically significant [51]. Oblique rotation was done because correlation coefficients of the components were > 0.3 [51]. Items were considered for deletion if cross-loadings were ≥ 0.3 [27] and loadings on any of the components < 0.4 [49, 52, 53]. To assess whether the data fits the established structure, confirmatory factor analysis (CFA) was performed. Goodness of fit was tested by using the comparative fit index (CFI), root mean square error of approximation (RMSEA), and standardized root mean square residual (SRMR). The following cut-off values were used for these measures: CFI ≥ 0.95, RMSEA ≤ 0.06, and SRMR ≤ 0.08 [27, 54]. Subsequently, presence of floor and ceiling effects were present if at least 15% of patients reported either the lowest or highest possible score on the total PSIT and subscales [55].

Reliability was measured by examining internal consistency and test-retest reliability. Internal consistency was assessed using Cronbach’s alpha coefficients (α) and values of at least 0.70 reflect satisfactory internal consistency [27]. Test-retest reliability was assessed by calculating the ICC (two-way mixed effects model, single measure) and should be at least 0.80 [27].

To examine construct validity, Spearman’s rho correlation coefficients were calculated between the PSIT subscales and the additional questionnaires. A priori, five hypotheses were formulated (Table 1). Instruments measuring a similar construct (i.e., convergent validity) should show an r ≥ 0.50, dissimilar but related constructs should show 0.30 > *r* < 0.50, and unrelated constructs should show *r* < 0.30 [27, 56]. Construct validity is considered to be good if ≥ 75% of the hypotheses are supported by the results, moderate if 50–75% of the hypotheses are supported, and poor if ≤ 50% of the hypotheses are supported [57].

**Table 1:** A priori formulated hypotheses to evaluate construct validity

No.	Hypothesis
1	Strong and positive correlations (r ≥ 0.50) were expected between PSIT subscale 1 and the PHQ-9, STAI-S, IES-R, and a strong and negative correlation (r ≥ − 0.50) between PSIT subscale 1 and domain 2 of the WHOQOL-Bref.
2	Strong and positive correlations (r ≥ 0.50) were expected between PSIT subscale 2 and the STAI-S, IES-R, and the PHQ-9.
3	A moderate and negative correlation (r ≥ −0.30 but < −0.50) was expected between PSIT subscale 2 and domain 1 of the WHOQOL-Bref.
4	Strong and negative correlations (r ≥ −0.50) were expected between PSIT subscale 3 and the RSES and domains 2 and 3 of the WHOQOL-Bref.
5	A moderate and negative correlation (r ≥ −0.30 but < −0.50) was expected between PSIT subscale 3 and domain 1 of the WHOQOL-Bref.

*Abbreviations*: *No.* Number, *PSIT* Psychosocial Screening Instrument for Trauma patients, *PHQ-9* Patient Health Questionnaire-9, *STAI-S* State-Trait Anxiety Inventory-State subscale, *IES-R* Impact of Events Scale-Revised, *WHOQOL-Bref* World Health Organization Quality of Life-Abbreviated Version, *RSES* Rosenberg Self-Esteem Scale

Receiver operating characteristics (ROC) analyses were performed to evaluate the ability of the PSIT to detect patients with psychosocial problems [58]. The area under the curve (AUC) should be at least 0.7 [27]. Furthermore, sensitivity, specificity, positive predictive value (PPV), and negative predictive value (NPV) were calculated for each potentially appropriate cut-off value, based on the ROC analyses. The most appropriate cut-off value corresponds with optimum sensitivity and specificity, which can be expressed by the Youden’s Index (J) [58]. J is a measure of diagnostic accuracy which can be calculated by the formula J = (sensitivity + specificity) - 1 [58]. CFA was conducted using IBM AMOS version 24. All other data analyses were done using IBM SPSS version 24.

## Results

### Patient characteristics

**Fig. 1:**
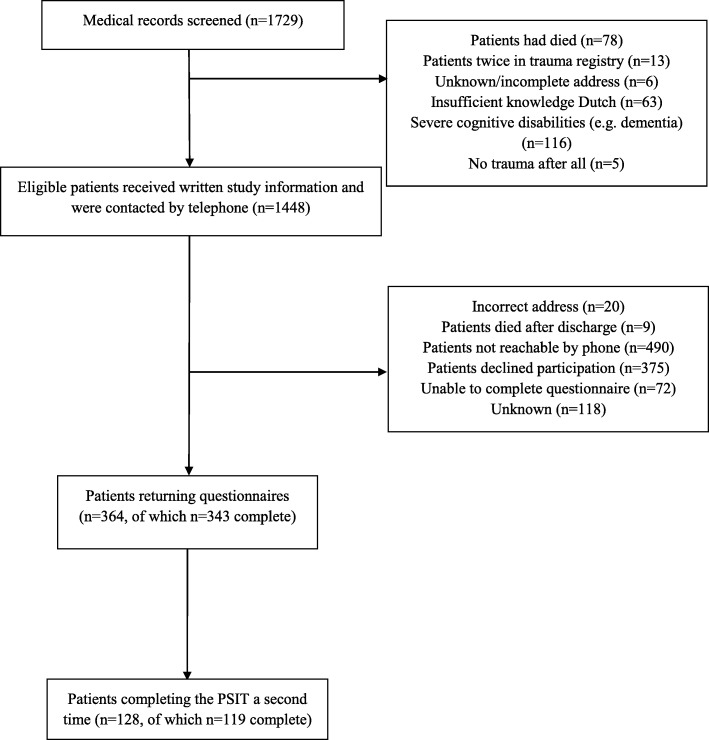
Flow chart of participant selection

The BTR database contained 1729 trauma patients admitted to the ETZ from October 2016 through September 2017. Patients were excluded if they had died (*n* = 78), had insufficient knowledge of the Dutch language (*n* = 63), had severe cognitive disorders such as dementia (*n* = 116), did not have an injury after all according to the electronical medical file (*n* = 5), or if their address was unknown or incomplete (*n* = 6). Furthermore, 13 patients were registered twice in the BTR database. In total, 1448 eligible patients were invited to participate of which 364 returned the questionnaires (response rate: 25.1%). The PSIT was completed a second time by 128 patients (response rate: 78.5%; Fig. 1). There was no difference between responders and non-responders regarding ISS (Median = 5 for both groups, Mann-Whitney U = 173,292, *p* = 0.14), gender (χ^2^ (1, *n* = 1448) = 0.43, *p* = 0.50), injury cause (χ^2^ (7, *n* = 1346) = 9.25, *p* = 0.24), and ICU admission (χ^2^ (1, *n* = 1448) = 1.20, *p* = 0.27; Table 2). However, responders were slightly older (Median = 64.4) compared to non-responders (Median = 62.0) (Mann-Whitney U = 181,211, *p* = 0.02) but this was a small effect (*r* = 0.06). In addition, patients with penetrating injury were less likely to respond, although the effect size was small (χ^2^ (1, *n* = 1448) = 5.95, *p* = 0.02, phi = −0.06). Table 3 presents the demographic and clinical characteristics of the patients in the total group and of the patients included in the test-retest analysis.

**Table 2:** Demographic and clinical characteristics of the responders and non-responders

	Responders (*n* = 364)	Non-responders (*n* = 1084)	Difference between responders and non-responders
	Median (IQR)	Median (IQR)	Mann-Whitney U (*p*-value)
Age at time of injury (years)	64.4 (52.0–78.0)	62.0 (41.0–77.0)	U = 181,211 (*p* = 0.02, *r* = 0.06)
ISS	5 (4–9)	5 (2–9)	U = 173,292 (*p* = 0.14)
Missing (n, %)	3 (0.8%)	71 (6.5%)	
	N (%)	N (%)	χ^2^ (*p*-value)
Gender			
Female	152 (41.8%)	474 (43.7%)	χ^2^ = 0.43 (*p* = 0.50)
Male	212 (58.2%)	610 (56.3%)	
ISS			
< 16	320 (87.9%)	993 (91.6%)	χ^2^ = 2.86 (*p* = 0.09)
≥ 16	41 (11.3%)	91 (8.4%)	
Missing	3 (0.8%)	0 (0.0%)	
Injury cause			
Falls	193 (53.0%)	548 (50.6%)	χ^2^ = 9.251 (*p* = 0.24)
Road traffic injury	108 (29.7%)	268 (23.7%)	
Work-related	24 (6.6%)	51 (4.7%)	
Sports-related	26 (7.1%)	60 (5.5%)	
Violence	5 (1.4%)	37 (3.4%)	
Intentional injury	3 (0.8%)	14 (1.3%)	
Other	1 (0.3%)	8 (0.8%)	
Missing	4 (1.1%)	98 (9.0%)	
Injury mechanism			
Blunt	358 (98.4%)	1029 (94.9%)	χ^2^ = 5.95 (*p* = 0.02, phi = −0.06)
Penetrating	6 (1.6%)	48 (4.4%)	
Missing	0 (0.0%)	7 (0.6%)	
ICU admission (yes)	61 (16.8%)	156 (14.4%)	χ^2^ = 1.2 (*p* = 0.27)

*Abbreviations*: *IQR* Interquartile range, *ISS* Injury Severity Score, *ICU* Intensive Care Unit

**Table 3:** Demographic and clinical characteristics of the patients

	Total group (*n* = 364)	Test-retest group (*n* = 128)
	Mean ± SD	Mean ± SD
Age at time of injury (years)	62.7 ± 17.3	64.4 ± 15.0
ISS	7.5 ± 6.5	8.5 ± 7.1
Time since injury (months)	7.9 ± 3.6	7.3 ± 3.7
Time between baseline and retest (days)		19.5 ± 6.8
	N (%)	N (%)
Gender		
Female	152 (41.8%)	59 (46.1%)
Male	212 (58.2%)	69 (53.9%)
Level of education		
Low	173 (47.5%)	57 (44.5%)
Middle	104 (28.6%)	38 (29.7%)
High	83 (22.8%)	30 (23.4%)
Unclassified	3 (0.8%)	0 (0%)
Missing	1 (0.3%)	3 (2.4%)
Current living situation		
Alone	109 (29.9%)	36 (28.1%)
With partner/family	255 (70.1%)	92 (71.9%)
Currently a paid job (yes)	136 (37.4%)	44 (35.4%)
Missing	1 (0.3%)	0 (0%)
ISS		
< 16	320 (87.9%)	107 (83.6%)
≥ 16	41 (11.3%)	21 (16.4%)
Missing	3 (0.8%)	0 (0%)
Injury cause		
Falls	193 (53.0%)	65 (50.8%)
Road traffic injury	108 (29.7%)	41 (32%)
Work-related	24 (6.6%)	5 (3.9%)
Sports-related	26 (7.1%)	8 (6.3%)
Violence	5 (1.4%)	1 (0.8%)
Intentional injury	3 (0.8%)	0 (0%)
Other	1 (0.3%)	1 (0.8%)
Missing	4 (1.1%)	7 (5.5%)
Injury mechanism		
Blunt	358 (98.4%)	125 (97.7%)
Penetrating	6 (1.6%)	3 (2.3%)
ICU admission (yes)	61 (16.8%)	24 (18.8%)
Pre-injury psychological problems (yes)	52 (14.3%)	15 (11.7%)
Pre-injury psychological treatment (yes)	51 (14.0%)	13 (10.2%)
Current psychological treatment (yes)	54 (14.8%)	23 (18%)

*Abbreviations ISS* Injury Severity Score, *ICU* Intensive Care Unit

### Internal structure

Initial PCA revealed three components with an Eigenvalue > 1, but there were several items with high cross-loadings which hampered interpretation of the structure. After an iterative process in which these items were deleted one by one and PCA was repeated, five items were deleted in the following order: ‘feelings of loneliness’, ‘problems with work/finances’, ‘feeling like a burden’, ‘excessive worrying’, and ‘more emotional’. The remaining 15 items loaded each on one component with loadings ≥ 0.4, thus revealing a simple and interpretable structure. The three components explained 64.5% of the variance and were labeled (1) Negative affect, (2) Anxiety and PTSS, and (3) Social and self-image (Table 4).

**Table 4:** Final results principal components analysis with oblique rotation^a^

Item	Content	Component 1: Negative affect	Component 2: Anxiety and PTSS	Component 3: Social and self-image
14	Anger	0.867		
11	Frustration	0.844		
12	Disappointment	0.839		
13	Feeling powerless	0.825		
10	Less social/leisure activities than desired	0.756		
15	Relationship	0.683		
2	Depressed mood	0.493		
7	Returning memories, nightmares, and/or flashbacks of the injury		0.853	
8	Feeling upset when thinking about the trauma		0.815	
1	Anxiety, feeling tensed		0.686	
9	Increased watchfulness		0.636	
3	Intimacy/sexual problems			0.887
4	Feeling less attractive			0.753
6	Decreased self-confidence			0.507
5	Inadequate social support			0.462

^a^Only factor loadings ≥ 0.4 are presented. *Abbreviations PTSS* Post-Traumatic Stress Symptoms

**Fig. 2:**
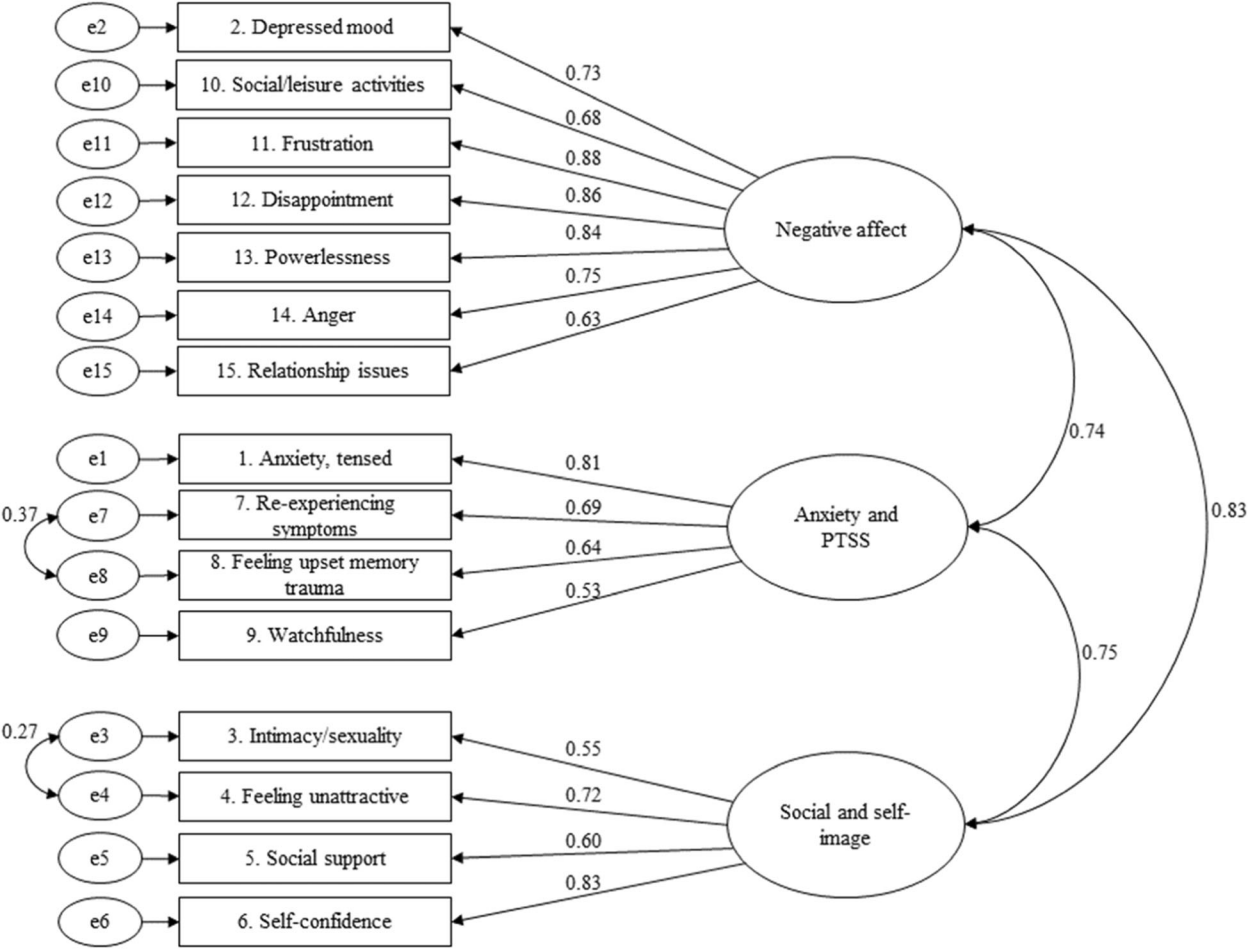
Final confirmatory factor model PSIT

Initial CFA revealed an acceptable model fit (χ^2^ (87) = 240.55, CFI = 0.95, RMSEA = 0.07, and SRMR = 0.05). To improve the model fit, two correlations of two error terms were added to the model (‘Intimacy/sexuality’ with ‘Attractiveness’; ‘Re-experiencing symptoms’ with ‘Feeling upset with memories’). This resulted in an excellent model fit (χ^2^ (85) = 191.58, CFI = 0.96, RMSEA = 0.06, and SRMR = 0.04) (Fig. 2). Additional file 1: Table S1 presents for each item of the final PSIT the missing rates, distribution of responses, kurtosis, and skewness. The final PSIT and its instructions are presented in Additional file 2.

### Reliability

A high Cronbach’s alpha was found for the total PSIT (15 items, α = 0.92), subscale 1 (Negative affect, 7 items, α = 0.91), subscale 2 (Anxiety and PTSS, 4 items, α = 0.77), and subscale 3 (Social and self-image, 4 items, α = 0.79) (Table 5). Patients completing the PSIT twice returned the second instrument on average within 19.5 ± 6.8 days. The ICC was 0.86 (95% confidence interval (CI) = 0.81–0.90), reflecting a good test-retest reliability.

**Table 5:** Cronbach’s alpha coefficients and floor and ceiling effects of the total PSIT and the subscales

	Possible min - max	Observed min - max	Median	IQR	Cronbach’s alpha	Floor (%)	Ceiling (%)
Total PSIT	0–45	0–42	5	2–13	0.92	9.9	0.0
Subscale 1: Negative affect	0–21	0–21	2	0–7	0.91	26.9	0.3
Subscale 2: Anxiety and PTSS	0–12	0–12	2	1–4	0.77	20.3	0.8
Subscale 3: Social and self-image	0–12	0–12	1	0–2	0.79	47.0	0.3

*Abbreviations*: *IQR* Interquartile Range, *PSIT* Psychosocial Screening Instrument for Trauma patients, *PTSS* Post-Traumatic Stress Symptoms

### Floor and ceiling effects

No ceiling effects were found (Table 5). Floor effects were observed for every subscale of the PSIT, namely 26.9% for Negative affect (minimum (min) - maximum (max): 0–21), 20.3% for Anxiety and PTSS (min - max: 0–12), and 47% for Social and self-image (min - max: 0–12). There were no floor effects regarding the total PSIT (9.9%) (min - max: 0–45).

**Table 6:** Spearman’s rho correlations coefficients between the subscales of the PSIT and between the PSIT and the additional questionnaires

	PSIT subscale 1: Negative affect	PSIT subscale 2: Anxiety and PTSS	PSIT subscale 3: Social and self-image
PSIT subscale 2: Anxiety and PTSS	0.58*		
PSIT subscale 3: Social and self-image	0.66*	0.50*	
PHQ-9	0.75*	0.59*	0.60*
STAI-S	0.66*	0.53*	0.55*
IES-R	0.66*	0.75*	0.52*
RSES	−0.50*	−0.32*	−0.49*
WHOQOL-Bref facet 1: Overall QoL and general health	−0.65*	−0.38*	−0.49*
WHOQOL-Bref Domain 1	−0.66*	−0.40*	−0.49*
WHOQOL-Bref Domain 2	−0.67*	−0.44*	−0.56*
WHOQOL-Bref Domain 3	−0.46*	−0.21*	−0.45*
WHOQOL-Bref Domain 4	−0.50*	−0.31*	−0.38*

*p<0.01 (two-tailed); Correlations in bold are as expected, underlined correlations are not as expected. *Abbreviations PSIT* Psychosocial Screening Instrument for Trauma patients, *PHQ-9* Patient Health Questionnaire-9, *STAI-S* State-Trait Anxiety Inventory-State subscale, *IES-R* Impact of Events Scale-Revised, *RSES* Rosenberg Self-Esteem Scale, *WHOQOL-Bref* World Health Organization Quality of Life-Abbreviated Version

### Construct validity

All correlations between the subscales of the PSIT and the additional questionnaires were statistically significant at the *p* < 0.01 level (Table 6). Ten of 12 correlations (83.3%) were as expected, confirming four of the five a priori formulated hypotheses (80%). This result indicates a good construct validity.

### ROC analyses and cut-off values

**Fig. 3:**
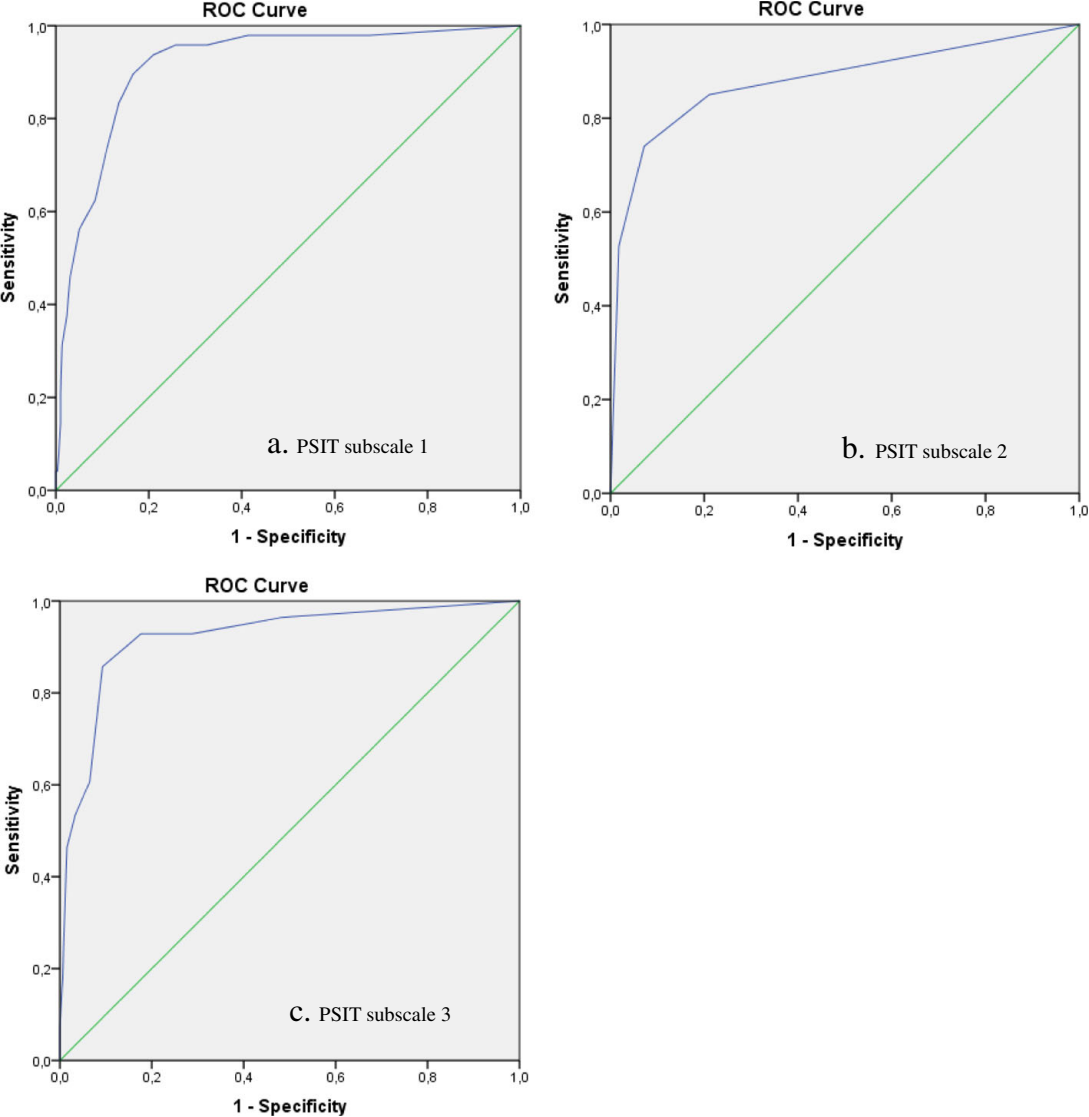
**a** Area Under the Curve (AUC) of subscale 1 of the PSIT versus the PHQ-9. **b** Area Under the Curve (AUC) of subscale 2 of the PSIT versus the STAI-S and IES-R. **c** Area Under the Curve (AUC) of subscale 3 of the PSIT versus the RSES

Figures 3a to c present the AUC curves for each subscale of the PSIT. Each scale has a high diagnostic accuracy showing an AUC of 0.92 for Negative affect (standard error = 0.02, 95%CI = 0.87–0.96, *p* < 0.01), 0.88 for Anxiety and PTSS (standard error = 0.02, 95%CI = 0.84–0.92, *p* < 0.01), and 0.92 for Social and self-image (standard error = 0.03, 95%CI = 0.86–0.98, *p* < 0.01). Table 7 shows per PSIT subscale the sensitivity, specificity, J, PPV, and NPV for each potential cut-off value. A cut-off score of 7 on Negative affect resulted in a sensitivity of 89.6% and a specificity of 83.4%; a cut-off value of 3 on Anxiety and PTSS showed a sensitivity of 94.4% and specificity of 90.3%; and a cut-off value of 4 on Social and self-image had a sensitivity of 85.7% and a specificity of 90.7%.

**Table 7:** Cut-off value analyses for each subscale of the PSIT

	Sensitivity	Specificity	J	PPV	NPV
Subscale 1: Negative affect					
5	0.958	0.742	0.700	0.377	0.991
6	0.938	0.79	0.728	0.421	0.987
**7**	**0.896**	**0.834**	**0.730**	**0.467**	**0.980**
8	0.833	0.864	0.697	0.500	0.970
9	0.729	0.892	0.621	0.522	0.953
10	0.625	0.915	0.54	0.953	0.938
Subscale 2: Anxiety and PTSS					
2	0.958	0.672	0.630	0.515	0.978
3	**0.944**	**0.903**	**0.846**	**0.779**	**0.978**
4	0.817	0.954	0.771	0.866	0.935
5	0.620	0.985	0.605	0.936	0.877
Subscale 3: Social and self-image					
2	0.929	0.712	0.641	0.218	0.991
3	0.929	0.824	0.753	0.313	0.992
**4**	**0.857**	**0.907**	**0.764**	**0.444**	**0.987**
5	0.607	0.935	0.542	0.447	0.965

Cut-off values with the highest J are in bold

*Abbreviations J* Youden’s Index, *PPV* Positive predictive value, *NPV* Negative predictive value, *PTSS* Post-Traumatic Stress Symptoms

### Feedback PSIT

Thirty-four patients (9.3%) reported that they found one of the questions in the PSIT confusing or difficult. One patient required assistance to complete the PIST, another patient found the item regarding re-experiencing symptoms ambiguous, and a third patient was confused regarding the difference between ‘frustration’ and ‘disappointment’. The most common remarks were that patients found the questions confronting (*n* = 7) and that some of the experienced problems were not related to the trauma (*n* = 5). In other words, only three patients had difficulty with interpreting one or more items of the PSIT. Therefore, it was decided that it was not needed to change the wording of the items or the response options.

Thirty patients (8.2%) stated that they missed a topic in the PSIT, most often related to physical or cognitive problems (*n* = 11) and less often to psychosocial problems (e.g., ‘feeling unhappy’, *n* = 2). Since the goal of the PSIT is to screen for psychosocial problems, the suggested topics were not included in the final PSIT. The optional open-ended question was retained to provide patients the opportunity to write an experienced problem not listed in the PSIT.

## Discussion

The aim of the current study was to finalize the PSIT, a recently developed psychosocial screening instrument for adults following physical trauma, and to examine its psychometric properties. After PCA and CFA, the final PSIT consists of 15 items covering three subscales and one optional open-ended question to provide patients the opportunity to report any other problem they might have (Additional file 2). This study indicates that the PSIT is an easy to complete, reliable and valid self-report psychosocial screening instrument. Less than 10% of patients indicated difficulties with one or more items, but this was most often related to finding the questions confronting and only three patients actually had difficulty with interpreting one or more items of the PSIT. This supports the notion that the PSIT is easy to complete and, therefore, no changes were made to the wording of the items and response options. In addition, few patients missed a topic in the PSIT. Suggestions for additional topics were most often related to physical or cognitive problems. As such problems can be reflections of psychosocial problems (e.g., concentration problems [59]), and the PSIT is intended to assess psychosocial problems, no additional items were included.

For nearly each item on the PSIT, missing values were below 2%. Only one item had a higher percentage of missing values, namely ‘relationship issues’ (3.8%). It is plausible that patients did not answer this item because they did not have a romantic relationship, since several patients had written down that they were single. Nevertheless, this missing rate (3.8%) is still far below the threshold of a problematic missing rate of 15% or more [27].

All subscales of the PSIT had floor effects. A disadvantage of floor effects is that discrimination between patients without psychosocial problems is not possible [27]. However, the PSIT is meant to result in the differentiation in patients who do and who do not experience psychosocial problems. Any attempt to discriminate within the group of patients without problems is not possible. Therefore, floor effects are not considered problematic [27].

As expected, strong correlations were found between the subscales of the PSIT. Research shows that psychosocial problems can be related or co-existing [60–62]. Consequently, it was expected that the scales of the PSIT would be interrelated. Nonetheless, PCA revealed a three-component structure with an excellent model fit as demonstrated by CFA.

Concerning the construct validity, only one hypothesis for the third subscale of the PSIT (Social and self-image) could not be confirmed. Moderate correlations were found between this subscale and the RSES and domain 3 of the WHOQOL-Bref, while high correlations were expected. This could be explained by the fact that this PSIT subscale contains items related to self-confidence and social problems and therefore measures a slightly broader construct than the other two instruments, which are focused on either self-esteem (the RSES [42]) or social relationships (domain 3 of the WHOQOL-Bref [46]).

The current study has some limitations. First, response bias might have occurred as only 25.1% of the eligible trauma patients responded to the questionnaire. Analyses revealed that younger age and penetrating injury were associated with being a non-responder, although effect sizes for these variables were small. Responders and non-responders were comparable on other characteristics (gender, ISS, ICU admission, injury cause). The majority of eligible patients were not reachable. Patients declining participation and willing to provide the reason often indicated that they were not interested because they were participants in other studies, they did not experience any psychosocial problems, or they found the questionnaire too long and/or burdensome. The response rate for the second PSIT (to assess test-retest reliability) was higher, namely 78.5%. This group completed the first questionnaire and was therefore already willing to participate in this study. Second, 63 patients were excluded based on their insufficient knowledge of the Dutch language. Yet, this is only 3.6% of the total trauma population, implying a relatively low risk for language or cultural bias.

Future research should explore whether the established cut-off values are useful in clinical practice and how the referral system could be organized. For instance, to whom should referral occur (e.g., psychologist, medical social work)? Another relevant research area is appropriate timing of psychosocial screening (e.g., 1 week, 2 months post-injury). Moreover, future studies might consider exploring how the PSIT can be best implemented in trauma care. Once these questions are addressed, the PSIT could be translated in different languages to assess its cross-cultural validity.

This study also has a number of clinical implications. While various questionnaires and screening instruments are available, these mainly assess depressive and anxiety symptoms (such as the Hospital Anxiety and Depression Scale [19]), or PTSS (such as the Impact of Events Scale [20]). The PSIT is the first psychosocial screening instrument for adult trauma patients which covers a range of all relevant psychosocial problems in one instrument. Although the literature increasingly advocates to monitor trauma patients’ wellbeing, the focus is primarily on depressive symptoms, post-traumatic stress symptoms, and anxiety symptoms [24]. The PSIT screens for these symptoms but also other psychological and social problems relevant to trauma patients. HCPs in trauma care now have a tool to systematically screen for psychosocial problems, which is short and easy to complete. The proposed cut-off values provide criteria by which patients should be referred for psychosocial aftercare.

## Conclusion

In conclusion, this study showed that the PSIT is a reliable, valid, and easy to complete psychosocial screening instrument. It appears to be a useful instrument to screen for psychosocial problems after injury.

## Supplementary information

**Additional file 1: Table S1.** Items of the PSIT, missing scores, distribution of responses, kurtosis, and skewness.

**Additional file 2.** The PSIT and its scoring instructions.

## Data Availability

Data supporting the findings of this study are available from the corresponding author upon reasonable request.

## Abbreviations

AUC: Area under the curve
BTR: Brabant Trauma Registry
CFA: Confirmatory factor analysis
CFI: Comparative fit index
CI: Confidence interval
HCP: Healthcare providers
ICC: Intraclass correlation coefficient
ICU: Intensive care unit
IES-R: Impact of events scale-revised
ISS: Injury severity score
J: Youden’s Index
KMO: Kaiser-Meyer-Olkin measure
Max: Maximum
Min: Minimum
NPV: Negative predictive value
PCA: Principal components analysis
PHQ-9: Patient health questionnaire-9
PPV: Positive predictive value
PSIT: Psychosocial screening instrument for trauma patients
PTSS: Post-traumatic stress symptoms
QoL: Quality of life
RMSEA: Root mean square error of approximation
ROC: Receiver operating characteristics
RSES: Rosenberg self-esteem scale
SCI: Spinal cord injury
SRMR: Standardized root mean squared residual
STAI-S: State-Trait Anxiety Inventory-State
TBI: Traumatic brain injury
WHOQOL-Bref: World Health Organization Quality of Life-Abbreviated version
