# Supplementary material for: Psychometric properties of the psychosocial screening instrument for physical trauma patients (PSIT)
Source: Health Qual Life Outcomes. 2019 Nov 12;17:172. doi: 10.1186/s12955-019-1234-6 (PMC6852899; doi:10.1186/s12955-019-1234-6)
Supplement: Supplementary file 1 — Additional file 1: Table S1. Items of the PSIT, missing scores, distribution of responses, kurtosis, and skewness. [file 12955_2019_1234_MOESM1_ESM.docx]

Additional file 1: Table S1. Items of the PSIT, missing scores, distribution of responses, kurtosis, and skewness

|  |  |  | **Distribution of responses (n, %)** | | | |  |  |
| --- | --- | --- | --- | --- | --- | --- | --- | --- |
| **Item** | **Content** | **Missings (n, %)** | **Not at all** | **A little** | **Quite a lot** | **Very much** | **Kurtosis** | **Skewness** |
| 1 | Anxiety, feeling tensed | 3 (0.8%) | 218 (59.9%) | 87 (23.9%) | 44 (12.1%) | 12 (3.3%) | 1.256 | 0.634 |
| 2 | Depressed mood | 3 (0.8%) | 196 (53.8%) | 116 (31.9%) | 35 (9.6%) | 14 (3.8%) | 1.206 | 0.830 |
| 3 | Sexual/intimacy problems | 5 (1.4%) | 264 (72.5%) | 59 (16.2%) | 27 (7.4%) | 9 (2.5%) | 1.913 | 2.944 |
| 4 | Feeling less attractive | 4 (1.1%) | 279 (76.6%) | 53 (14.6%) | 24 (6.6%) | 4 (1.1%) | 2.095 | 3.778 |
| 5 | Inadequate social support | 3 (0.8%) | 276 (75.8%) | 57 (15.7%) | 21 (5.8%) | 7 (1.9%) | 2.152 | 4.199 |
| 6 | Decreased self-confidence | 4 (1.1%) | 226 (62.1%) | 89 (24.5%) | 33 (9.1%) | 12 (3.3%) | 1.447 | 1.358 |
| 7 | Returning memories, nightmares, and/or flashbacks of the injury | 3 (0.8%) | 256 (70.3%) | 77 (21.2%) | 17 (4.7%) | 11 (3.0%) | 1.975 | 3.620 |
| 8 | Feeling upset when thinking about the trauma | 4 (1.1%) | 270 (74.2%) | 62 (17.0%) | 19 (5.2%) | 9 (2.5%) | 2.128 | 4.156 |
| 9 | Increased watchful | 4 (1.1%) | 105 (28.8%) | 132 (36.3%) | 90 (24.7%) | 33 (9.1%) | 0.373 | -0.809 |
| 10 | Less social/leisure activities than desired | 5 (1.4%) | 199 (54.7%) | 81 (22.3%) | 55 (15.1%) | 24 (6.6%) | 1.026 | -0.139 |
| 11 | Frustration | 5 (1.4%) | 209 (57.4%) | 86 (23.6%) | 38 (10.4%) | 26 (7.1%) | 1.245 | 0.464 |
| 12 | Disappointment | 4 (1.1%) | 198 (54.4%) | 99 (27.2%) | 48 (13.2%) | 15 (4.1%) | 1.095 | 0.260 |
| 13 | Feeling powerless | 3 (0.8%) | 193 (53.0%) | 98 (26.9%) | 43 (11.8%) | 27 (7.4%) | 1.097 | 0.146 |
| 14 | Anger | 4 (1.1%) | 235 (64.6%) | 78 (21.4%) | 37 (10.2%) | 10 (2.7%) | 1.465 | 1.280 |
| 15 | Relationship issues | 14 (3.8%) | 273 (75.0%) | 50 (13.7%) | 22 (6.0%) | 5 (1.4%) | 2.180 | 4.228 |

Abbreviations: PSIT=Psychosocial Screening Instrument for Trauma patients.
