# Supplementary material for: Psychometric properties of the psychosocial screening instrument for physical trauma patients (PSIT)
Source: Health Qual Life Outcomes. 2019 Nov 12;17:172. doi: 10.1186/s12955-019-1234-6 (PMC6852899; doi:10.1186/s12955-019-1234-6)
Supplement: Supplementary file 2 — Additional file 2. The PSIT and its scoring instructions. [file 12955_2019_1234_MOESM2_ESM.docx]

Additional file 2: The PSIT and its scoring instructions

Instruction: Below is a list of several problems which can be experienced by physical trauma patients. Please state to what extent you experienced each problem during the past week. Please check one of the four answers. There is no right or wrong answer, we are interested in your experience.

|  |  | **Not at all** | **A little** | **Quite a lot** | **Very much** |
| --- | --- | --- | --- | --- | --- |
| 1 | Anxiety, feeling tensed | 🞎 | 🞎 | 🞎 | 🞎 |
| 2 | Depressed mood | 🞎 | 🞎 | 🞎 | 🞎 |
| 3 | Problems with intimacy/sexuality | 🞎 | 🞎 | 🞎 | 🞎 |
| 4 | Feeling less attractive | 🞎 | 🞎 | 🞎 | 🞎 |
| 5 | Inadequate social support | 🞎 | 🞎 | 🞎 | 🞎 |
| 6 | Decreased self-confidence | 🞎 | 🞎 | 🞎 | 🞎 |
| 7 | Recurring memories, nightmares, and/or images (flashbacks) of the trauma | 🞎 | 🞎 | 🞎 | 🞎 |
| 8 | Feeling upset when thinking about the trauma | 🞎 | 🞎 | 🞎 | 🞎 |
| 9 | Increased watchfulness | 🞎 | 🞎 | 🞎 | 🞎 |
| 10 | Less social/leisure activities than desired | 🞎 | 🞎 | 🞎 | 🞎 |
| 11 | Frustration | 🞎 | 🞎 | 🞎 | 🞎 |
| 12 | Disappointment | 🞎 | 🞎 | 🞎 | 🞎 |
| 13 | Feeling powerless | 🞎 | 🞎 | 🞎 | 🞎 |
| 14 | Anger | 🞎 | 🞎 | 🞎 | 🞎 |
| 15 | Relationship issues | 🞎 | 🞎 | 🞎 | 🞎 |

Do you experience other psychosocial problems than those listed above? If so, please describe them below and state to what extent you experienced this problem in the past week?

|  |  | **Not at all** | **A little** | **Quite a lot** | **Very much** |
| --- | --- | --- | --- | --- | --- |
| 16 | Other problem or problems, namely:  ………………………………….……………… | 🞎 | 🞎 | 🞎 | 🞎 |

Scoring instructions
Items 1-15 can be scored 0 (not at all), 1 (a little), 2 (quite a lot), or 3 (very much).
Subscale 1 (Negative affect): Sum items 2, 10, 11, 12, 13, 14, and 15. Cut-off value ≥ 7 indicative for mood disturbances.
Subscale 2 (Anxiety and PTSS): Sum items 1, 7, 8, and 9. Cut-off value ≥ 3 indicative for anxiety symptoms and/or PTSS.
Subscale 3 (Social and self-image): Sum items 3, 4, 5, and 6. Cut-off value ≥ 4 indicative for social or sexual problems, negative body image, and/or decreased self-confidence.
Item 16 is optional and does not belong to a subscale.
